# Augmentation strategy fluoxetine and lurasidone in the treatment of OCD with comorbid Restrictive Anorexia: a case report

**DOI:** 10.1192/j.eurpsy.2022.1658

**Published:** 2022-09-01

**Authors:** L. Orsolini, S. Bellagamba, S. Tempia Valenta, V. Salvi, U. Volpe

**Affiliations:** 1 Unit of Clinical Psychiatry, Polytechnic University of Marche, Ancona, Italy, Department Of Neurosciences/dimsc, Ancona, Italy; 2 Polytechnic University of Marche, Department Of Clinical Neurosciences/dimsc, School Of Medicine, Unit Of Psychiatry, Ancona, Italy; 3 Unit of Clinical Psychiatric, Polytechnic University of Marche, Ancona, Italy, Department Of Neurosciences/dimsc, Ancona, Italy

**Keywords:** obsessive-compulsive disorder, Anorexia nervosa, lurasidone

## Abstract

**Introduction:**

Obsessive-Compulsive Disorder (OCD) is characterized by the presence of intrusive thought (obsessions) and ritualistic behaviour (compulsions). First-choice psychopharmacological treatment is based on serotonin reuptake inhibitors (SRIs). However, about half of OCD do not or partially respond to SRIs (TR-OCD) and need an augmentation strategy with second-generation antipsychotics (SGAs).

**Objectives:**

We report a case of severe OCD with comorbid anorexia nervosa, restrictive type (AN-r) treated with fluoxetine (up to 40 mg daily) and lurasidone (37 mg daily bedtime) augmentation.

**Methods:**

At baseline and monthly 4-months-follow-up were administered Y-BOCS-II (Yale-Brown Obsessive Compulsive Scale), CGI-S (Clinical Global Impression-Severity), SCL-90 (Symptom Checklist-90 items) and EDI-3 (Eating Disorder Inventory-3).

**Results:**

Compared to the baseline, a clinically significant clinical response was observed on OC at Y-BOCS-II (≥35% Y-BOCS reduction) and eating symptomatology at EDI-3 after 1 month of augmentation treatment, while a full remission after 3 months (Y-BOCS scoring ≤ 14)(p<0.01). We also noticed, throughout clinical follow up interviews, improvement in patient’s social skills and life satisfaction.

**Conclusions:**

Further longitudinal and real-world effectiveness studies are needed to confirm these preliminary findings and investigate the potential of lurasidone augmentation strategy in attenuating OC symptomatology in TR-OCD and whereas a comorbid AN-r is present.

**Disclosure:**

No significant relationships.

